# Some Japanese mothers do not follow package instructions of infant formula: a web-based analytical cross-sectional study

**DOI:** 10.1186/s40795-022-00615-7

**Published:** 2022-11-01

**Authors:** Kaori Endoh

**Affiliations:** Laboratory of Public Health Nutrition, Department of Food Sciences and Nutrition, Kyoritsu Women’s University, 2-2-1 Hitotsubashi, Chiyoda-ku, 101-8437 Tokyo, Japan

**Keywords:** Infant formula, Feeding behavior, Attitude to health, Mothers, Diet

## Abstract

**Background:**

Not following the infant formula package instruction endangers infant health. Although infant formula misuse has been reported abroad, its incidence in Japan remains unknown. Furthermore, it is reasonable to assume that experience in childcare reduces the likelihood of making mistakes in using infant formula. This study aimed to examine the association between compliance with infant formula package instruction and childcare experience in Tokyo and surrounding prefectures in Japan.

**Methods:**

Using a web-based questionnaire, mothers with infants were analyzed cross-sectionally and surveyed regarding their infants’ nutrition and formula preparation methods in August 2021. Compliance with the infant formula package was determined according to (a) using unlabeled infant formula, (b) preparing infant formula without reading package instructions, (c) giving formula to children ≥ 2 h after preparing, and (d) adding other ingredients to the formula bottle. The association between the misuse of infant formula and childcare experience was examined by grouping the participants by infant age (< 6 months and ≥ 6 months), and by comparing first-born child status with later-born. Of the 333 mothers with infants, 3 were excluded due to out-of-scope responses, and 330 were included in the analysis.

**Results:**

The major sources of information on infant feeding methods among the participants were obstetric facilities (92.1%), internet (36.1%), and family (20.9%). The proportions of participants using infant formulas not labeled as “infant formula,” such as follow-up milk, not preparing at prescribed concentrations, feeding infant formulas > 2 h after preparation, and adding additional ingredients to the bottle were 7.9%, 4.1%, 23.1%, and 15.9%, respectively, which suggest the misuse of infant formula. These four answers did not differ significantly between mothers of children aged < 6 months and ≥ 6 months or between those with first-born and later-born children.

**Conclusion:**

This study suggested that some Japanese mothers do not follow package instructions of infant formula in Japan. The misuse of infant formula may not be related to the length of time spent in childcare or the presence or absence of childcare experience. Providing appropriate information on the correct use of infant formula to all caregivers, regardless of their parenting experience, is required.

**Supplementary Information:**

The online version contains supplementary material available at 10.1186/s40795-022-00615-7.

## Background

The international food standard for labelling indicates that instructions for proper use should also be included [1]. It is reported that the labeling on infant formula packages is beyond the comprehension of the average consumer [2] and caregivers may be preparing formula without reading the package instructions or understanding them. In a systematic review that did not include Japanese reports, at least 11 studies showed that formula was given to children without being prepared according to the prescribed concentrations [3]. Observational studies on formula feeding have reported the addition of cereal to baby bottles in the USA [4]. Moreover, in Brazil, formulas prepared by parents of lower socioeconomic status contained less protein and fat, suggesting that the prepared formula was diluted more than prescribed (i.e., with lower than prescribed concentrations of the formula) [5]. Infants who were fed with formula prepared at a concentration higher than prescribed developed hypernatremia requiring medical treatment in Taiwan [6]. It is suggested that one of the reasons of bottle-fed infants rapidly gaining weight is the inaccurate weighing of infant formula [7]. An increase in infant formula intake at 3 months of age was associated with an increase in systolic and diastolic blood pressure in adulthood [8], and rapid weight gain in childhood led to future obesity [9].

Caregivers who feed formula to their infants find it difficult to consult medical professionals because breastfeeding is often strongly encouraged and promoted as the best form of nutrition for infants [10]. For this reason, caregivers might rely on others’ experience of feeding practice by consulting friends and family or obtaining information from the internet. It is assumed that inexperienced caregivers expect that a longer and more frequent experience in childcare comes with a better understanding of childcare, including the preparation of infant formula. However, health information on the internet may not always be accurate [11, 12] and may lead to a misuse of infant formula. To prevent adverse effects of infant formula, the WHO/Food and Agriculture Organization guideline on how to prepare formula for bottle-feeding at home recommends using hot water at 70 °C or higher to prepare infant formula [13]. It also states that adding more or less powdered milk than directed may cause the infant to become ill, and that infant formula should be discarded after 2 h of being prepared.

The prevalence of breastfeeding at 6 months in Japan is comparable to that in Australia and the Republic of Korea [14]. However, legal regulations on formulas, follow-up formulas, and infant formulas differ by country. In Japan, two types of formula are available: (1) infant formula for infants aged < 1 year, which can be used as a replacement for breast milk, and (2) follow-up milk for children aged > 1 year with insufficient nutrient intake (Table 1). Making the right choice of infant formula in Japan requires checking whether it is labeled as “Foods for Special Dietary Uses,“ a label approved by the Consumer Affairs Agency. Infant formula is classified as a “Food for Special Dietary Uses” under the Health Promotion Act [15] and must contain a specified amount of energy and nutrients for infants. However, unlike “Foods for Special Dietary Uses,“ follow-up milk is treated as a general food type with no legal standards or restrictions. It may not be easy for caregivers to distinguish between infant formula and follow-up milk [16]. While infant formula contains the essential nutrients for infants covered by the Health Promotion Act [15], the non-standardization of follow-up milk results in varying amounts of energy and nutrients across manufacturers. Feeding infants with follow-up milk may result in malnutrition or overfeeding.

**Table 1:** Classification of infant formula in Japan [29]

	Regulations	Age of infants	
Infant formulas	Foods for Special Dietary Uses under the Health Promotion Act	< 1 year old	・Infant formula in powdered form ・Infant formula in liquid form
Follow-up milk	Foods in General	> 1 year old	

Since the breastfeeding rate and infant formula use in Japan are similar to those reported in other countries, the possibility of infant formula misuse exists. This may include thinner or thicker concentrations than prescribed, giving infants aged < 1 year follow-up milk instead of infant formula, adding substances such as cereal, sugar to the feeding bottle or using water < 70 °C to prepare infant formula. Caregivers hesitate to consult medical professionals about infant formula. Alternatively, they rely on the internet and family or friends’ experiences. Health information on the internet [11, 12] or the experience of family or friends is not always correct. Examining the relationship between appropriate feeding methods and parenting experience may show that feeding methods information on internet or the experience of family or friends might not be always reliable. Therefore, this study focused on the relationship between feeding method and the length of time spent in childcare or the presence or absence of childcare experience. This web-based survey aimed to determine whether caregivers (a) used infant formula and not follow-up milk, (b) prepared infant formula at prescribed concentrations, (c) fed their children infant formula within 2 h of being prepared, and (d) did not added sugar or other ingredients to feeding bottles. To examine whether childcare experience is an influencing factor in the ability to correctly prepare and offer infant formula, the results were analyzed by child age group (< 6 months and ≥ 6 months), and first-born status (as compared with later-born).

## Methods

This web-based analytical cross-sectional survey was conducted by the internet research company Macromill, Inc. (Tokyo, Japan) from August 16 to 19, 2021. Since regional differences in the sale status of food products exist, participation was limited to residents of Tokyo and surrounding prefectures (Kanagawa, Chiba, and Saitama). Since females are the primary caregivers in Japan, the study participants were limited to mothers. Infant are defined under one year of age under Maternal and Child Health Law in Japan [17], and mothers with children under one year of age were included in this study.

Questionnaire items included the following: information sources on feeding methods, concerns about feeding, feeding methods (i.e., breastfeeding, infant formula, combined breastfeeding and infant formula, and other), source of the infant formula (location of purchase), and whether the infant formula was prepared and fed according to the package. The questions used in this study are presented in Additional file 1; no other information was collected. The survey was based on the National Nutrition Survey on Preschool Children conducted by the Ministry of Health, Labor, and Welfare in 2015 [18].

After survey completion, data were received from Macromill for analysis. According to the Guide to Support Feeding and Weaning in Japan [19], complementary food typically commences at approximately 5–6 months of age. The participants were divided into two age groups: participants with children aged < 6 months and those with children aged ≥ 6 months. In addition, participants were subdivided into two groups, those with a first-born and those with later-born, based on their responses to breastfeeding differences in childcare experiences. The proportions of those in the four different groups were then calculated. Chi-square test or Fisher’s exact probability method was used to test the difference between the < 6 months and ≥ 6 months groups and between the first-born and later-born groups. The Fisher exact probability test was used if the expected value was less than 5, and the chi-square test was used if the expected value was greater than 5. Questions with multiple answers were excluded from statistical analyses (questions 4 and 6 in Additional file 1). A *p*-value of < 0.05 was considered significant. To evaluate the relationship between the misuse of infant formula and childcare experience, logistic regression analysis was conducted, and odds ratio and 95% confidence intervals were calculated. Dependent variables were as follows: using infant formula labeled as infant formula, preparing infant formula as instructed on the labels, not giving formula to children > 2 h after preparation, and not added something to the feeding bottle. The independent variables were as follows: “the age of the child (< 6 months or ≥ 6 months)”, and “first child or other than first child”. The crude model was used. Statistical analyses were performed using IBM SPSS Statistics version 25 (IBM Corp, Armonk, NY, USA) and the R-based free statistical software EZR [20, 21].

## Ethical considerations

An explanatory document with the contact details of the principal investigator was displayed on the screen. Before answering the questionnaire, the participants were informed of the main purpose of the study; that identifiable personal information, including name, address, and e-mail address, would not be collected to minimize the risk of data leaks; and that participation in the study could not be canceled after their consent was provided. Only those who understood the purpose of the study and were willing to participate were allowed to proceed to the questionnaire screen. A confirmation screen was set up to ascertain whether the participants provided informed consent, and only those who had given consent proceeded to the questionnaire screen. The address of the response form was sent to the panel registered with Macromill. The participants could decide whether to answer or not to answer any of the questions.

## Results

**Fig. 1:**
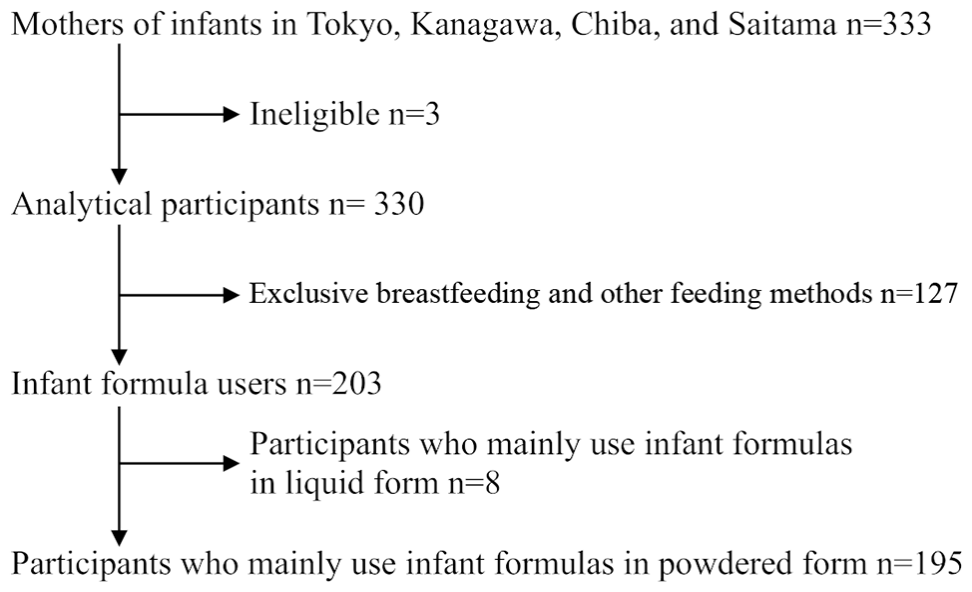
Flow diagram showing the flow of response

A total of 333 registered monitors of the internet research company who matched the criteria of mothers with infants living in Tokyo and surrounding prefectures of Kanagawa, Chiba, and Saitama responded to the survey. Among the respondents, three women were excluded: one participant with 12 children and two participants with children who were already weaned. Thus, 330 participants were included in the analysis. As the analysis was not limited to infant formula users, the responses for infant formula were analyzed using the procedure presented in Fig. 1. The proportion of participants according to age-specific nutrition methods by months is shown in Table 2. The overall proportions of breastfeeding mothers, infant formula users, combined breastfeeding mothers and infant formula users, and those using other methods were 38.2%, 24.5%, 37.0%, and 0.3%, respectively.

**Table 2:** Proportions of breast milk, infant formula, and breast milk/infant formula consumption by age (months)^a^

		Age (months)										
	**All participants**	**1**	**2**	**3**	**4**	**5**	**6**	**7**	**8**	**9**	**10**	**11**
Breast milk	38.2 (126)	57.1 (12)	23.3 (7)	42.5 (17)	44.7 (17)	36.1 (13)	58.4 (14)	33.3 (10)	40.0 (14)	32.1 (9)	21.8 (5)	32.0 (8)
Infant formula	24.5 (81)	4.8 (1)	10.0 (3)	17.5 (7)	15.8 (6)	25.0 (9)	20.8 (5)	40.0 (12)	40.0 (14)	25.0 (7)	39.1 (9)	32.0 (8)
Breast milk /infant formula	37.0 (122)	38.1 (8)	63.4 (19)	40.0 (16)	39.5 (15)	38.9 (14)	20.8 (5)	26.7 (8)	20.0 (7)	42.9 (12)	39.1 (9)	36.0 (9)
Other	0.3 (1)	0 (0)	3.3 (1)	0 (0)	0 (0)	0 (0)	0 (0)	0 (0)	0 (0)	0 (0)	0 (0)	0 (0)

^**a**^The numbers in parentheses represent the proportions of total participants (n = 330)

Table 3 shows the breastfeeding status of the participants. The major source of information on infant feeding was obstetric facilities, followed by the internet and family. The feeding method (i.e., breastfeeding, infant formula, combined breastfeeding and infant formula, and others) was significantly different by child age (< 6 months and ≥ 6 months) or first-born and later-born status.

**Table 3:** Information on feeding (all participants)^a^

	All		Child age				First-born status		
	(n = 330)		< 6 months (n = 165)	≥ 6 months (n = 165)	*p* value		First-born (n = 174)	Later-born (n = 156)	*p* value
Is the child you are currently feeding your first-born?									
First-born	52.7 (174)^b^		44.2 (73)^c^	61.2 (101)^d^	0.002^*^				
Later-born	47.3 (156)		55.8 (92)	38.8 (64)					
Where did you get the information about feeding your child? (Multiple answers allowed)									
(1) Obstetric facility	92.1 (304)		89.7 (148)	94.5 (156)	-		93.7 (163)^e^	90.4 (141)^f^	-
(2) Pediatric facilities	10.0 (33)		7.3 (12)	12.7 (21)			10.3 (18)	9.6 (15)	
(3) Nursery	3.9 (13)		3.0 (5)	4.8 (8)			5.7 (10)	1.9 (3)	
(4) Local antenatal classes	8.2 (27)		7.9 (13)	8.5 (14)			8.6 (15)	7.7 (12)	
(5) Infant health check up	10.0 (33)		6.1 (10)	13.9 (23)			10.9 (19)	9.0 (14)	
(6) Family	20.9 (69)		16.4 (27)	25.5 (42)			24.7 (43)	16.7 (26)	
(7) Friends	10.0 (33)		10.3 (17)	9.7 (16)			10.9 (19)	9.0 (14)	
(8) Internet	36.1 (119)		32.7 (54)	39.4 (65)			40.8 (71)	30.8 (48)	
(9) Others	6.4 (21)		4.2 (7)	8.5 (14)			6.3 (11)	6.4 (10)	
Which of the following concerns do you have about feeding your child? (Multiple answers allowed)									
(1) I do not know if I am providing enough breast milk or infant formula (powdered or liquid milk)	48.8 (161)		46.7 (77)	50.9 (84)	-		55.7 (97)	41.0 (64)	-
(2) Insufficient or no breast milk production	22.1 (73)		26.7 (44)	17.6 (29)			21.3 (37)	23.1 (36)	
(3) The child refusing to drink infant formula (powdered or liquid milk)	12.4 (41)		10.9 (18)	13.9 (23)			11.5 (20)	13.5 (21)	
(4) The child drinking too much infant formula (powdered or liquid form)	7.9 (26)		7.3 (12)	8.5 (14)			7.5 (13)	8.3 (13)	
(5) The child does not want to drink breast milk	5.2 (17)		3.6 (6)	6.7 (11)			5.7 (10)	4.5 (7)	
(6) The child drinks too much breast milk	7.9 (26)		10.3 (17)	5.5 (9)			6.9 (12)	9.0 (14)	
(7) No place to breastfeed when you go out	27.6 (91)		32.7 (54)	22.4 (37)			28.7 (50)	26.3 (41)	
(8) The child is not gaining weight steadily	8.2 (27)		4.2 (7)	12.1 (20)			10.3 (18)	5.8 (9)	
(9) Children gain too much weight	11.2 (37)		12.1 (20)	10.3 (17)			11.5 (20)	10.9 (17)	
(10) I do not know when or how to stop feeding	26.1 (86)		18.2 (30)	33.9 (56)			37.9 (66)	12.8 (20)	
(11) Mother is not in good health	3.9 (13)		2.4 (4)	5.5 (9)			5.2 (9)	2.6 (4)	
(12) The mother’s work schedule prevents her from breastfeeding as much as she would like	2.4 (8)		3.0 (5)	1.8 (3)			1.7 (3)	3.2 (5)	
(13) I do not have anyone I can talk to about breastfeeding	7.6 (25)		6.7 (11)	8.5 (14)			10.3 (18)	4.5 (7)	
(14) Others	10.3 (34)		8.5 (14)	12.1 (20)			6.9 (12)	14.1 (22)	
Which of the following methods do you currently use to feed your child?									
(1) Breast milk	38.2 (126)		40.0 (66)	36.4 (60)	0.001^*^		35.1 (61)	41.7 (65)	0.026^*^
(2) Infant Formula	24.5 (81)		15.8 (26)	33.3 (55)			31.0 (54)	17.3 (27)	
(3) Breast milk/infant formula	37.0 (122)		43.6 (72)	30.3 (50)			33.9 (59)	40.4 (63)	
(4) Other	0.3 (1)		0.6 (1)	0 (0)			0 (0)	0.6 (1)	

^a^The numbers in parentheses represent the proportions of total participants

^b^The percentage of participants (n = 330)

^c^The proportion of those with children  < 6 months of age (n = 165)

^d^The proportion of participants with children aged ≥ 6 months (n = 165)

^e^The proportion of those with a first-born (n = 174)

^f^The proportion of participants with later-born children (n = 156)

^*^p < 0.05

Information on the use of prepared infant formula (answers from infant formula users and combined breast milk/infant formula users only) is presented in Table 4. The overall proportion of unlabeled infant formula users was 7.9%, and the proportion did not differ significantly by child age (< 6 months and ≥ 6 months) or first-born and later-born status. The forms of infant formula (i.e., powdered or liquid) only differed significantly by first-born and later-born status. The proportion of main purchasers of infant formula differed significantly only by child age (< 6 months and ≥ 6 months).

**Table 4:** Information on the use of prepared infant formula (infant formula and breast milk/infant formula users)^a^

	All		Child age					First-born status			
	(n = 203)		< 6 months (n = 98)	≥ 6 months (n = 105)	*p* value				First-born (n = 113)	Later-born (n = 90)	*p* value
Does the infant formula you use have the label “Infant formula in powdered form " or “Infant formula in liquid form “?											
(1) Yes	92.1 (187)^b^		91.8 (90)^c^	92.4 (97)^d^	0.886				94.7(107)^e^	88.9 (80)^f^	0.128
(2) No	7.9 (16)		8.2 (8)	7.6 (8)					5.3(6)	11.1 (10)	
Which of the following do you use most often?											
(1) Infant formula in powdered form	96.1 (195)		96.9 (95)	95.2 (100)	0.534				99.1(112)	92.2 (83)	0.012^*^
(2) Infant formula in liquid form	3.9 (8)		3.1 (3)	4.8 (5)					0.9(1)	7.8 (7)	
Where do you buy most of your infant formula?											
(1) Supermarket	6.4 (13)		6.1 (6)	6.7 (7)	0.086				7.1(8)	5.6 (5)	0.309
(2) Drug stores and pharmacies	53.7 (109)		52.0 (51)	55.2 (58)					47.8(54)	61.1 (55)	
(3) Online shop	28.1 (57)		34.7 (34)	21.9 (23)					31.9(36)	23.3 (21)	
(4) Others	11.8 (24)		7.1 (7)	16.2 (17)					13.3(15)	10.0 (9)	
Who among the following has chosen to use infant formula?											
(1) Mother	94.1 (191)		91.8(90)	96.2 (101)	0.468				92.9 (105)	95.6 (86)	0.695
(2) Father	3.4 (7)		4.1 (4)	2.9 (3)					3.5(4)	3.3 (3)	
(3) Family	0.5 (1)		1.0 (1)	0 (0)					0.9(1)	0 (0)	
(4) Friends	0 (0)		0 (0)	0 (0)					0(0)	0 (0)	
(5) Health professionals	2.0 (4)		3.1 (3)	1.0 (1)					2.7(3)	1.1 (1)	
(6) Others	0 (0)		0 (0)	0 (0)					0(0)	0 (0)	
Who among the following is most likely to purchase infant formula?											
(1) Mother	75.9 (154)		68.4 (67)	82.9 (87)	0.042^*^				70.8(80)	82.2 (74)	0.160
(2) Father	20.2 (41)		25.5 (25)	15.2 (16)					24.8(28)	14.4 (13)	
(3) Family	3.9 (8)		6.1 (6)	1.9 (2)					4.4(5)	3.3 (3)	
(4) Friends	0 (0)		0 (0)	0 (0)					0(0)	0 (0)	
(5) Others	0 (0)		0 (0)	0 (0)					0(0)	0 (0)	

^a^The numbers in parentheses represent the proportions of total participants

^b^All participants who answered for infant formula and breast milk/infant formula (n = 203)

^c^The proportion of infants aged < 6 months whose mothers answered infant formula and breast milk/infant formula (n = 98)

^d^The proportion of those  ≥ 6 months of age whose mothers answered infant formula and breast milk/infant formula (n = 105)

^e^The proportion of mothers with a first-born, who answered for infant formula and breast milk/infant formula (n = 113)

^f^The proportion of mothers with children later-born, who answered for infant formula and breast milk/infant formula (n = 90)

Information on prepared infant formula use (responses from those who used infant formula in powdered form only) is shown in Table 5. The overall proportions of those who did not use a measuring spoon and the prescribed amount of hot water to prepare the infant formula according to the directions on the package, who gave their children infant formula that had been prepared > 2 h before feeding, and participants who added “something” to the infant formula and fed it to their children were 4.1%, 23.1%, and 15.9%, respectively. These items were not significantly different by child age (< 6 months and ≥ 6 months) or first-born and later-born status. No significant association was observed between the misuse of infant formula and childcare experience (Table 6).

**Table 5:** Information on the use of prepared infant formula (only powdered infant formula users)^a^

	All		Child age				First-born status		
	(n = 195)		< 6 months (n = 95)	≥ 6 months (n = 100)	*p* value		First-born (n = 112)	later-born (n = 83)	*p* value
Do you use a measuring spoon and the prescribed amount of hot water to make infant formula according to the directions on the package?									
(1) Yes	95.9 (187)^b^		96.8 (92)^c^	95.0 (95)^d^	0.517		94.6 (106)^e^	97.6 (81)^f^	0.305
(2) No	4.1 (8)		3.2 (3)	5.0 (5)			5.4 (6)	2.4 (2)	
Have you ever given your child infant formula that has been made more than two hours after you made?									
(1) Yes	23.1 (45)		27.4 (26)	19.0 (19)	0.166		22.3 (25)	24.1 (20)	0.771
(2) No	76.9 (150)		72.6 (69)	81.0 (81)			77.7 (87)	75.9 (63)	
Have you ever added anything to your infant formula and given it to your child?									
(1) Yes	15.9 (31)		16.8 (16)	15.0 (15)	0.725		16.1 (18)	15.7 (13)	0.938
(2) No	84.1 (164)		83.2 (79)	85.0 (85)			83.9 (94)	84.3 (70)	

^a^The numbers in parentheses represent the proportions of total participants

^b^The proportion of all participants who used infant formula in powdered form (n = 195)

^c^The proportion of infants <6 months old who were fed with infant formula in powdered form (n = 95)

^d^The proportion of those  ≥ 6 months old who were fed with infant formula in powdered form (n = 100)

^e^The proportion of participants with a first-born who used infant formula in powdered form (n = 112)

^f^The numbers in this row represent the proportion of participants with later-born children, who answered for infant formula and breast milk/infant formula (n = 83)

^*^p < 0.05

**Table 6:** Relationship between misuse of infant formula and child care experience

		Child age (< 6 months old / ≥ 6 months old)			First-born status (First-born/Later-born)	
		OR (95% CI)	*p* value		OR (95%CI)	*p* value
Does the infant formula you use have the label “Infant formula in powdered form " or “Infant formula in liquid form “?	Yes	Reference			Reference	
	No	0.928 (0.334–2.580)	0.886		2.230 (0.778–6.390)	0.136
Do you use a measuring spoon and the prescribed amount of hot water to make infant formula according to the directions on the package?	Yes	Reference	.		Reference	
	No	1.610 (0.375–6.950)	0.520		0.436 (0.086–2.220)	0.317
Have you ever given your child infant formula that has been made more than two hours after you made?	Yes	Reference			Reference	
	No	1.610 (0.819–3.150)	0.168		0.905 (0.463–1.770)	0.771
Have you ever added anything to your infant formula and given it to your child?	Yes	0.871 (0.404–1.880)	0.725		0.970 (0.446–2.110)	0.938
	No	Reference			Reference	

Identifiable individual information was contained in the answers to free-response questions (questions 3, 5, 7, 9, 13, 15, 17, and 21 in Additional file 1). Thus, the results of the free-response questions were excluded from the analysis.

## Discussion

This study examined the relation between compliance with infant formula package instruction and childcare experience in Tokyo and its surroundings using a web-based questionnaire. When questioned about using milk not labeled as infant formula, not preparing at prescribed concentrations, giving infant formula > 2 h after preparation, and adding “something” to the bottle, the results suggested that some caregivers do not follow infant formula package instructions in Japan. These four answers were not different by child age (< 6 months and ≥ 6 months) or first-born and later-born status. Therefore, infant formula misuse might not be related to childcare experience in Japan.

The proportion of using unlabeled infant formulas was 7.9%. In Japan, infant formulas must be labeled as “Foods for Special Dietary Uses” under the Health Promotion Act [15], and it is possible that the participants were mistakenly feeding follow-up milk to their infants instead. In Italy, advertising of infant formula is not permitted, while advertising of follow-on milk is permitted. Nevertheless, caregivers of infants answered that they have seen advertisements for infant formula [16]. Unlike infant formula, follow-up milk is labeled as “Foods in General” and does not have nutritional regulations in Japan. Feeding follow-up milk to infant may not provide infants with the nutrients they need or may have excessive nutritional content for infants, and these lead to future elevated blood pressure [8] or obesity [9]. As it may be difficult for consumers to differentiate between infant formula and follow-up milk [16], it is necessary to provide appropriate information that will lead to correct product selection.

Preparing infant formula without reading the package instruction was reported by 4.1% of the study participants. Not adjusting concentrations as specified may lead to a risk of undernutrition or overnutrition, and inaccurate weighing of formula may be the reason why bottle-fed infants gain weight faster than breastfed infants [7]. According to the guidelines for safe preparation, storage, and handling of powdered infant formula detailed by the WHO [13], infant formula packages should state that a clean bottle should be used, the hands of the person preparing the infant formula should be clean, the formula should be prepared at the specified concentration using the measuring spoon provided, and hot water > 70 °C. These precautions might help avoid adverse effects, such as *Cronobacter sakazakii* contamination which resulted in the death of two infants in the United States [22, 23]. Understandably, infant formula is labeled with safety information in Japan. Infants infected with *Cronobacter sakazakii* can develop serious disorders, and since cases have been reported in Japan [24] there may be infants at risk because instructions on packages are not being followed. The precautions presented by WHO guidelines are often displayed with illustration on the packaging of infant formula. Nevertheless, some caregivers do not follow these instructions on the package. The risks of not using infant formula appropriately as shown on the package might lead to prevent inappropriate use of infant formula.

Poor compliance with infant formula instructions by Japanese mothers included (a) using follow-up milk instead of infant formula (7.9%), (b) not preparing at the prescribed concentrations (4.1%), (c) giving formula to children > 2 h after preparation (23.1%), and (d) adding “something” to the formula before giving it to the child (15.9%). The length and experience of childcare may not be influencing factors for the correct preparation and feeding of infant formula. In Japan, prepared milk for infants should be labeled with the statement “It is appropriate to use with consultation and guidance from doctors and dietitians.” If doctors and dietitians become aware of the misuse of infant formula and follow-up milk, they can provide appropriate advice to caregivers regardless of the number of months post-delivery and childcare experience. Instructions should be revised for clarity to ensure that caregivers prepare powdered infant formula correctly, as previous research showed that the advertisements of formula were difficult to understand [16]. Immediately after childbirth, caregivers may not have time to check the instructions on infant formula packages because they are too busy [6]. The feeding methods followed for the first child are often continued in the same way for the second and subsequent children [25, 26]. Thus, if caregivers acquire appropriate childcare knowledge after the birth of their first child, they will be able to provide better childcare to subsequent infants. In this study, obstetric facilities were the primary source about feeding, but not many mothers might continuously attend obstetric facilities during the feeding period as they did before delivery. Next to obstetric facilities, internet and family were the most common sources of information on feeding, and however health information on internet is not always correct [11, 12]. Providing appropriate information on infant feeding and the opportunity to consult with medical professionals about breastfeeding and infant formula feeding via the internet, the second most useful source of information on breastfeeding for the participants of the present study, might help solve these problems.

This study is the first to reveal that Japanese mothers sometimes use infant formula incorrectly, regardless of the length or experience of childcare. This study also had some limitations. First, the study sample size was small because the included areas were limited to Tokyo and Saitama, Chiba, and Kanagawa prefectures, and participants who did not have internet access could not participate in this study; nevertheless, the number of participants was comparable to that in a previous study [27]. A large-scale and nationwide survey in combination with face-to-face surveys may be required to confirm the factors related to the misuse of infant formula. Second, this was an analytical cross-sectional study, and a cause-and-effect relationship could not be established between infant formula misuse and the length or experience of childcare, which requires further prospective research. Third, information about the participants’ socioeconomic status was not obtained because most individuals in Japan are reluctant to reveal information about their income. Forth, this study was conducted using a self-reported questionnaire that has not been validated.

## Conclusion

This study confirmed the existence of infant formula misuse among Japanese mothers, including using follow-up milk instead of infant formula, not preparing infant formula at the specified concentration, feeding formula > 2 h after preparation, and adding other substances to the feeding bottle. It is suggested that infant formula misuse may not be related to the length or experience of childcare. Therefore, it is necessary to provide appropriate information on the correct use of infant formula to caregivers to protect children’s health.

## Electronic supplementary material

Below is the link to the electronic supplementary material.

Supplementary Material 1: Supplemental figure1 Questionnaire items, Description of data: Questionnaire items

## Data Availability

The datasets are not publicly available due to ensure individual anonymity. Data are available from corresponding author on reasonable request for scientific purpose and with approval by the Research Ethics Review Committee of Kyoritsu Women’s University and Kyoritsu Women’s Junior College.

## List of abbreviations

WHO: World Health Organization
