# Supplementary material for: Some Japanese mothers do not follow package instructions of infant formula: a web-based analytical cross-sectional study
Source: BMC Nutr. 2022 Nov 1;8:126. doi: 10.1186/s40795-022-00615-7 (PMC9628175; doi:10.1186/s40795-022-00615-7)
Supplement: Supplementary Material 1: — Supplemental figure1 Questionnaire items, Description of data: Questionnaire items [file 40795_2022_615_MOESM1_ESM.doc]

1. How many months old is the child you are currently breastfeeding? Select one from the following.

(1) Under 1 month old

(2) Less than 1 to 2 months old

(3) Less than 2 to 3 months old

(4) Less than 3 to 4 months old

(5) Less than 4 to 5 months old

(6) Less than 5 to 6 months old

(7) Less than 6 to 7 months old

(8) Less than 7 to 8 months old

(9) Less than 8 to 9 months old

(10) Less than 9 to 10 months old

(11) Less than 10 to 11 months old

(12) Less than 11-12 months old

2. Is the child you are currently breastfeeding your first child?

(1) Yes

(2) No

3. If you answered "No" to question 2: If you don’t mind, tell us how many children you have, including those you are currently breastfeeding (free answer).

4 Where did you get the information about feeding your child? (Multiple answers allowed)

(1) Obstetric facility

(2) Pediatric facilities

(3) Nursery

(4) Pregnancy classes at the local government

(5) Infant health checkup at local government

(6) Family

(7) Friends

(8) Internet

(9) Others

5. If you answered "(9) Others" in question 4: If you don’t’ mind, answer where you got information about breastfeeding method (free answer).

6. Which of the following concerns do you have about breastfeeding? (Multiple answers are acceptable)

(1) I don't know if I'm getting enough breast milk or infant formula (powdered or liquid milk).

(2) Insufficient or no breast milk production

(3) the child refusing to drink infant formula (powdered or liquid milk)

(4) the child drinking too much infant formula (powdered or liquid form)

(5) The child does not want to drink breast milk

(6) The child drinks too much breast milk

(7) No place to breastfeed when you go out.

(8) The child is not gaining weight steadily.

(9) Children gain too much weight.

(10) I don't know when or how to stop feeding.

(11) Mother is not in good health.

(12) The mother's work schedule prevents her from breastfeeding as much as she would like

(13) I don't have anyone I can talk to about breastfeeding.

(14) Others

7. Those who answered "(14) Others" in question 6: If you don't mind, tell us about any problems you have with breastfeeding (free answer).

8. Which of the following methods do you currently use to feed your child?

(1) Breast milk

(2) Infant Formula

(3) Breast milk/Infant Formula

(4) Other

9. For those who answered "(4) Others" in question 8: If you don't mind, answer how you feed your child (free answer).

If you answered (1) or (4) to question 8, this is the end of the question. After this, only those who answered (2) or (3) in Question 8 answer.

10. Does the infant formula you use have the label "Infant formula in powdered form " or "Infant formula in liquid form "?

(1) Yes

(2) No

11. Which of the following do you use most often?

(1) Infant formula in powdered form

(2) Infant formula in liquid form

12. Where do you buy most of your infant formula?

(1) Supermarket

(2) Drug stores and pharmacies

(3) Internet

(4) Others

13. For those who answered "(4) Others" in question 12: If you don't mind, answer where you buy the products (free answer).

14. Who among the following has chosen to use infant formula?

(1) Mother of child

(2) Father of child

(3) Family

(4) Friends

(5) Health professionals

(6) Others

15. For those who answered "(6) Others" in question 14: If you don't mind, answer your choice of infant formula (free answer).

16. Who among the following is most likely to purchase infant formula?

(1) Mother of child

(2) Father of child

(3) Family

(4) Friends

(5) Others

17. For those who answered "(5) Others" in question 16: If you don't mind, answer who you buy infant formula from most often (free answer).

If you answered (2) in question 11, this is the end of the question. After this, answer only if you answered (1) in Question 11.

18. Do you use a measuring spoon and the prescribed amount of hot water to make infant formula according to the directions on the package?

(1) Yes

(2) No

19. Have you ever given your child infant formula that has been made more than two hours after you made?

(1) Yes

(2) No

20 Have you ever added anything to your infant formula and given it to your child?

(1) Yes

(2) No

21. If you answered "(1) Yes" to question 20: If you don’t mind, tell us what you added to your infant formula (Free answer).
